# Awareness of headache and of national headache society activities among primary care physicians – a qualitative study

**DOI:** 10.1186/1756-0500-6-118

**Published:** 2013-03-26

**Authors:** Andreas R Gantenbein, Christian Jäggi, Mathias Sturzenegger, Claudio Gobbi, Gabriele S Merki-Feld, Mark J Emmenegger, Ethan Taub, Peter S Sándor

**Affiliations:** 1 Neurorehabilitation, RehaClinic Bad Zurzach, Quellenstrasse 34, Bad Zurzach, CH-5330, Switzerland; 2 Headache and Pain Unit, Department of Neurology, University Hospital Zurich, Zurich, Switzerland; 3 IMK Institute for Medicine and Communication, Basel, Switzerland; 4 University Hospital Inselspital, Bern, Switzerland; 5 Neurocenter of Southern Switzerland, Lugano, Switzerland; 6 University Women’s Hospital Zurich, Zurich, Switzerland; 7 Neurological Practice, Agno, Switzerland; 8 Department of Neurosurgery, University Hospital Basel, Basel, Switzerland; 9 Neurorehabilitation, RehaClinic, Baden, Switzerland

**Keywords:** Headache, Primary care, MRI, Awareness, National society, Migraine

## Abstract

**Background:**

Headache is one of the most common symptoms in primary care. To improve the quality of headache diagnosis and management with the largest possible benefit for the general population, headache and pain societies around the world have recently been devoting more attention to headache in primary care.

The aim of the study was to investigate the potential contribution that national societies can make toward raising the awareness of primary headaches in general practice.

**Findings:**

In a qualitative telephone survey, targeting primary care practices (PCP), we asked about the frequency of headache patients in their practices and inquired about their treatment and referral strategies.

A total of 1000 telephone interviews with PCP have been conducted. Three-hundred and fifty physicians have been directly interviewed, 95% of them see headache patients every week, 23% daily. Direct MRI referral is done by 84%. Sixty-two per cent of the physicians knew the Swiss headache society, 73% were interested in further education about headaches.

**Conclusion:**

The survey yielded information about the physicians’ awareness of the Swiss Headache Society and its activities, and about their desire for continuing education in the area of headache. National headache societies should work to improve the cooperation between headache specialists and PCP, aiming for a better care for our patients with headache.

## Background

Although headache is one of the most common symptoms in primary care [1,2], the quality of headache diagnosis and treatment still leaves room for improvement [3,4]. In response to this need the International Headache Society (IHS) recently initiated a Primary Care Interest Group to promote improvement of the clinical management of migraine and headache [5]. What could be the additional contribution of the national societies? The Swiss Headache Society (SHS) was founded in 1995 to bring together medical specialists, primary care physicians, and scientists with a special interest in headache. Its goals are to promote headache research, improve the diagnosis and treatment of headaches, foster cooperation between physicians and other health-care providers, and provide education for both patients and physicians. Among other activities, the SHS publishes its therapeutic recommendations every two years in the form of a brochure, holds annual national meetings, and maintains an internet-based information platform for patients and physicians.

Studies looking at the effect of international and national societies on the behaviour of primary care physicians are limited. There is some research looking at the interaction of primary care physicians with national and international guidelines [6,7]. However, an extensive literature search did not reveal any data relating to the awareness of national (headache) societies by general practitioners. We conducted a survey targeting primary care practices (PCP) in the German- and French-speaking parts of Switzerland. The main topics addressed were the physicians’ caseload of headache patients; their behaviour in dealing with such patients with respect to treatment, referrals to specialists, and direct ordering of magnetic resonance imaging (MRI); and furthermore their interest in pursuing continuing medical education about headache, both in general and, specifically, from the SHS.

## Methods

This qualitative study and the questionnaire (see Table 1) were designed by a group of SHS members (the SHS Study Group). Structured telephone interviews involving a computer-assisted interview program and a standardized script were conducted by an experienced local company (amPuls Call Center, Luzern, Switzerland) from 29 October to 17 November 2010. Each interview took approximately 5 minutes. In a first step, the addresses and telephone numbers of 5314 primary care practices were retrieved from the national telephone directory. The total number of registered PCP in Switzerland in 2010 was 7638, according to the published statistics of the Swiss Medical Association [8]. Potential interview partners were selected at random until telephone interviews had been obtained from a targeted total of 1000 PCPs. When reaching the target, 784 PCP’s phone line was busy or disconnected, 824 further appointments would have been available, and 2706 PCPs declined: 1469 general rejection, 613 ‘no interest’, 408 ‘no time’, 216 other reason (language problem, no compensation etc.). Every participant gave oral, but no written consent, to take part in the interview. The selected physicians were contacted in their practices by a call to the fixed-line office telephone; if two attempts to contact the interviewee failed, his or her practice assistant was asked to answer the questions. The SHS Study Group received fully anonymized data for further analysis. The study was approved by the ethical committee of Zurich (KEK), Switzerland.

**Table 1:** The following questions and response options were included in the questionnaire


1. “How often do you see patients presenting with (any type of) headache as the main symptom?”					
once a day	> once a week	once a week	once in three months	never	don’t know
2. “Do you actively ask about headache?”					
yes	rarely	never			
3. “Do you treat patients with primary headache disorders?”					
yes	no	don’t know			
4. “Do you refer headache patients to a specialist?”					
always	yes, if treatment is not effective	rarely	don’t know		
5. “Do you refer patients directly for MRI?”					
yes	no	don’t know			
6. “Do you know the Swiss Headache Society?”					
yes	no	don’t know			
7. “Do you know about the following activities of the SHS?”					
treatment guidelines	national meeting	homepage	GP teaching afternoon		
8. “Are you interested in education offered by the SHS?”					
yes	no	don’t know			
9. “Which type of education would you be interested in?”					
headache refreshers	practice-based workshops	treatment of special headaches	discussion of own cases	other	
10. “Are you interested in receiving information on headache from the Swiss Headache Society by email?”					
yes	no	don’t know			
11. “Are you interested in a service kit containing treatment guidelines and flyers for patients?”					
yes, by postmail	yes, by e-mail	no	don’t know		

Fixed response categories were provided, always including the option “don’t know / no statement.”

## Findings

### Response rate and demographics

A total of 1000 interviews with PCP have been conducted, in 350 cases directly with the physician. Of the 1000 primary care practices from which interviews were obtained, 357 were run by internists and 643 by general practitioners (GPs). And 670 were in the German-speaking part of Switzerland, 330 in the French-speaking part. Of the 350 physicians 269 (77%) were male, 119 (34%) were internists and 231 (66%) were GPs.

### Caseload and treatment behaviour

**Figure 1:**
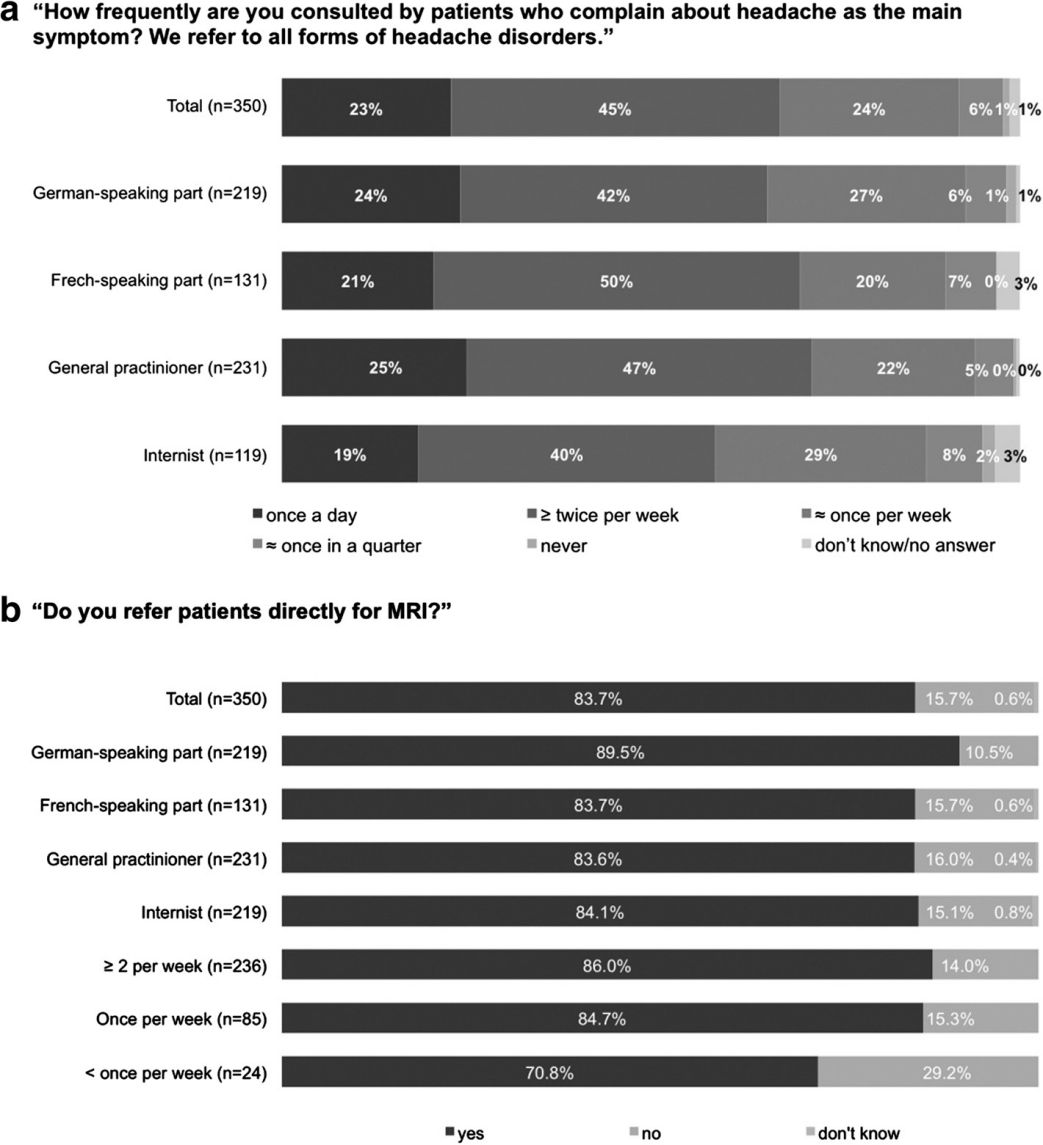
**The responses to two of the questions are shown below.** Panel (**a**) shows the frequency of headache in primary care practice: a difference is seen between GPs and internists. Panel (**b**) shows the large percentage of primary care physicians who refer their headache patients directly for MRI, with no difference between GPs and internists.

Among the 350 physicians, 92% reported that they saw at least one headache patient (i.e., a patient whose main symptom is headache) at least once per week. 23% (25% of GPs and 10% of internists) reported seeing such patients every day, 45% (47% and 40%) at least twice a week, and 24% once a week (see Figure 1).

Two-thirds of the physicians reported asking their patients actively about headache. On this item, there was a regional difference between the German- and French-speaking parts of Switzerland (53% vs. 92%), as well as a difference between specialties (74% of internists vs. 65% of GPs).

Of all the 1000 PCP surveyed, 86% reported treating patients with primary headaches (no reply to this question, 4%); the corresponding figure among the 350 physicians was 91% (no reply, 1%). Physicians who were acquainted with the SHS were slightly more likely to treat primary headache patients than those who were not (94% vs. 87%).

### Specialist referral and imaging

Only a very small percentage of the directly interviewed physicians (3%) said that they would refer any patient presenting to them with headache to a specialist. On the other hand, 31% of them said they would rarely or never refer a headache patient to a specialist, even in case of treatment failure (the corresponding figure for all practices surveyed was 26%). Many physicians said they ordered MRI scans for their headache patients themselves (84% of both internists and GPs; percentage of all 1000 practices, 80%) (see Figure 1).

### Awareness of the Swiss headache society and its activities

Of the 350 physicians who were directly interviewed 216 (62%) said they were acquainted with the Swiss Headache Society (SHS). This figure included 73% of the 219 German-speaking physicians, but only 44% of the 131 French-speaking physicians. 72% of the physicians who knew about the SHS (45% of all directly interviewed physicians) knew about the SHS’s treatment recommendations, 54% (33%) knew about its annual national meeting, 40% (25%) were familiar with its homepage (http://www.headache.ch), and 35% (21%) knew about its afternoon continuing medical education sessions for general practitioners.

### Interest in continuing medical education and information from the SHS

A total of 255 physicians (73%) expressed an interest in further education about headache, preferring the following topics: “headache refresher” (88%), “practice-oriented workshops and case reports” (85%), “treatment of rare headaches” (76%), and “discussion of own cases with experts” (48%).

Half of the physicians gave their e-mail addresses so that they could receive further information. A total of 72% of all practices (76% of the directly interviewed physicians) ordered the service pack by mail (including treatment recommendations and homepage flyers for patients), while only 2% (1%) chose to download the material from the website instead.

## Discussion

We were pleasantly surprised to find that 62% of the primary care physicians were acquainted with the Swiss Headache Society, but we still consider this figure too low from the point of view of a national society. However, there might be a bias in regard of PCPs agreeing to the interview, having some interest in headache and/or knowing the Swiss Headache Society. Many physicians reported that they avoided referring their headache patients to specialists - more than 20% said they did not treat headache patients at all. Of course, most patients with primary headaches can receive adequate treatment in primary care if they are not too severely affected and if good clinical guidelines are available; also, very experienced primary care physicians may need to call on their specialist colleagues less often than others. Nonetheless, in a study conducted in the United Kingdom, Kernick et al. reported that 70% of patients presenting to GPs with new-onset primary headaches did not receive a diagnosis [3], which might be due to the fact that primary healthcare often deals with early undifferentiated stages of illnesses. An earlier study in a Swiss tertiary care centre revealed a similarly high number of incorrect or unspecified diagnoses [9]. The clinical problem of headache demands appropriate medical attention, and it seems that the cooperation between primary care physicians and headache specialists still needs to be improved.

The large percentage of primary care physicians who said they themselves ordered MRI scans for their headache patients (over 80%) was also an unexpected finding, with potential implications for health-care costs. In an earlier Swiss case-control-study, far fewer imaging studies were ordered than this figure would suggest (16% of patients in general practice had a CT scan, 4.4% an MRI scan) [10]. Moreover, a recent, small-scale study from the U.K. about direct access to MRI in a primary care setting [11] led to the conclusion that GPs who know the correct indications for MRI can appropriately select patients with chronic headaches for neuroimaging, without any loss of diagnostic accuracy, and with the potentially significant benefits of earlier diagnosis, more appropriate onward referral, and even, in the end, lower costs. Patients also seemed to appreciate being referred for an MRI by their GP without needing to see a specialist first. No study, however, has yet addressed the potential harm done by incidental findings in neuroimaging studies that are performed on headache patients without a proper indication.

We found that primary care physicians want more information about headache, especially if it is directly applicable in everyday practice, yet few of them were interested in actively downloading such information from the Swiss Headache Society’s homepage (though half gave us their e-mail address so that we could send them more information electronically). The physicians seemed rather passive, compared to patients: a recent Japanese study revealed that most headache patients would consult websites for information - a greater percentage than would consult health professionals directly [12].

## Conclusion

Our survey yielded information on primary care physicians’ awareness of headache. We were surprised by the high percentages of PCPs who ordered MRIs directly and who did not refer their headache patients to specialists. These findings suggest that our national headache society and perhaps those in other countries as well, should work to improve the cooperation between headache specialists and primary care providers, with the goal of better care for patients with headache.

## Competing interests

All authors are members of the Swiss Headache Society, other than that they report no conflict of interest with the above study and manuscript.

This study was supported by unrestricted grants from Pfizer, MSD, and AstraZeneca. 

## Authors’ contributions

All authors, being part of the SHS study group, have made substantial contributions to conception and design, as well as analysis and interpretation of data; AG, CJ & PS have been mainly involved in the drafting of the manuscript. All authors have been revising the manuscript critically, and have given final approval of the version to be published.

## References

[B1] Bigal ME Bordini CA Speciali JG Etiology and distribution of headaches in two Brazilian primary care units Headache 2000 40 3 241 247 10.1046/j.1526-4610.2000.00035.x 10759928

[B2] Latinovic R Gulliford M Ridsdale L Headache and migraine in primary care: consultation, prescription, and referral rates in a large population J Neurol Neurosurg Psychiatry 2006 77 3 385 387 16484650 10.1136/jnnp.2005.073221 PMC2077680

[B3] Kernick D Stapley S Hamilton W GPs’ classification of headache: is primary headache underdiagnosed? Br J Gen Pract 2008 58 547 102 104 10.3399/bjgp08X264072 18307854 PMC2233960

[B4] MacGregor EA Brandes J Eikermann A Migraine prevalence and treatment patterns: the global Migraine and Zolmitriptan Evaluation survey Headache 2003 43 1 19 26 10.1046/j.1526-4610.2003.03004.x 12864754

[B5] Kernick D Reducing the burden of headache: The International Headache Society Primary Care Interest Group Cephalalgia 2010 30 8 899 901 20656699 10.1177/0333102409361216

[B6] Cabana MD Rand CS Powe NR Wu AW Wilson MH Abboud PA Rubin HR Why don’t physicians follow clinical practice guidelines? A framework for improvement JAMA 1999 282 15 1458 1465 10.1001/jama.282.15.1458 10535437

[B7] Lesho EP Myers CP Ott M Winslow C Brown JE Do clinical practice guidelines improve processes or outcomes in primary care? Mil Med 2005 170 3 243 246 15828703 10.7205/milmed.170.3.243

[B8] Swiss Medical Association Physicians statistics 2010 [http://www.fmh.ch/files/pdf5/2._Berufsttige_rzte_nach_Hauptfachgebiet_2010_d1.pdf]

[B9] Kozak S Gantenbein AR Isler H Merikangas KR Angst J Gamma A Agosti R Nosology and treatment of primary headache in a Swiss headache clinic J Headache Pain 2005 6 3 121 127 10.1007/s10194-005-0166-1 16355292 PMC3451640

[B10] Gantenbein AR Kozak S Agosti F Agosti R Isler H Headache patients in primary care and a tertiary care unit in Zurich, Switzerland Cephalalgia 2006 26 12 1451 1457 10.1111/j.1468-2982.2006.01225.x 17116095

[B11] Taylor TR Evangelou N Porter H Lenthall R Primary care direct access MRI for the investigation of chronic headache Clin Radiol 2012 67 1 24 27 10.1016/j.crad.2011.02.006 22088325

[B12] Imai N Yagi N Konishi T Serizawa M Kobari M Websites offer helpful information concerning consultation with headache specialists Cephalalgia 2010 30 4 496 499 19515123 10.1111/j.1468-2982.2009.01915.x

